# Observation of Weyl fermions in a magnetic non-centrosymmetric crystal

**DOI:** 10.1038/s41467-020-16879-1

**Published:** 2020-07-03

**Authors:** Daniel S. Sanchez, Guoqing Chang, Ilya Belopolski, Hong Lu, Jia-Xin Yin, Nasser Alidoust, Xitong Xu, Tyler A. Cochran, Xiao Zhang, Yi Bian, Songtian S. Zhang, Yi-Yuan Liu, Jie Ma, Guang Bian, Hsin Lin, Su-Yang Xu, Shuang Jia, M. Zahid Hasan

**Affiliations:** 1 Laboratory for Topological Quantum Matter and Advanced Spectroscopy (B7), Department of Physics, Princeton University, Princeton, NJ 08544 USA; 2 International Center for Quantum Materials, School of Physics, Peking University, Peking, China; 3 Rigetti Computing, Berkeley, CA 94720 USA; 4 Key Laboratory of Artificial Structures and Quantum Control, School of Physics and Astronomy, Shanghai Jiao Tong University, Shanghai, China; 5 Department of Physics and Astronomy, University of Missouri, Columbia, MO USA; 6 Institute of Physics, Academia Sinica, Taipei, 11529 Taiwan; 7 Collaborative Innovation Center of Quantum Matter, 100871 Beijing, China; 8 Princeton Institute for Science and Technology of Materials, Princeton University, Princeton, NJ 08544 USA; 9 Lawrence Berkeley National Laboratory, Berkeley, CA 94720 USA

**Keywords:** Theory and computation, Condensed-matter physics

## Abstract

The absence of inversion symmetry in non-centrosymmetric materials has a fundamental role in the emergence of a vast number of fascinating phenomena, like ferroelectricity, second harmonic generation, and Weyl fermions. The removal of time-reversal symmetry in such systems further extends the variety of observable magneto-electric and topological effects. Here we report the striking topological properties in the non-centrosymmetric spin-orbit magnet PrAlGe by combining spectroscopy and transport measurements. By photoemission spectroscopy below the Curie temperature, we observe topological Fermi arcs that correspond to projected topological charges of ±1 in the surface Brillouin zone. In the bulk, we observe the linear energy-dispersion of the Weyl fermions. We further observe a large anomalous Hall response in our magneto-transport measurements, which is understood to arise from diverging bulk Berry curvature fields associated with the Weyl band structure. These results establish a novel Weyl semimetal phase in magnetic non-centrosymmetric PrAlGe.

## Abstract (web summary)

The search for magnetic Weyl fermion remains a challenge. Here, the authors report angle-resolved photoemission spectroscopy and magnetotransport measurements resolving the topological properties of Weyl fermion quasiparticles in magnetic non-centrosymmetric crystal PrAlGe.

## Introduction

Development in the search for materials with topological electronic properties has rapidly progressed in the past decade. With a refined understanding of the role symmetries have on the electron wavefunctions Berry curvature, the experimental study of new and exotic quantum phenomena has now become widely accessible^1–18^. A well-recognized example is the breaking of time-reversal symmetry in magnetic materials, which may result in producing Berry curvature fields that generate an intrinsic anomalous Hall response. Along similar lines, the breaking of inversion symmetry in non-centrosymmetric materials is understood to be essential for fostering new quantum phenomena, such as non-local gyrotropic effects^19^, quantum nonlinear Hall effects^20^, photogalvanic effects^21^, accidental two-fold band degeneracies (Weyl fermions) that are protected by a quantized non-zero integer Chern number (chiral charge)^22–25^, and anomalous transport^26^.

In this study, we observe that magnetic non-centrosymmetric PrAlGe^14^ hosts the emergent topological properties of Weyl fermions by photoemission-based spectroscopy and magneto-transport. In contrast to previous works on magnetic Weyl semimetal candidates Mn_3_Sn^27,28^ and Co_3_Sn_2_S_2_^29–31^ (both centrosymmetric), PrAlGe is calculated to exhibit Weyl fermions in proximity to the Fermi level, making it more suitable for experimentally probing its Berry curvature properties and exploring the connection between photoemission-based band structure and transport. In addition, because PrAlGe lacks both inversion and time-reversal symmetry it can uniquely induce quantum spin currents without a concomitant charge current^14,15^. Motivating future studies on PrAlGe, we experimentally resolve the key topological properties of Weyl fermions by relying predominately on our measurements^11,32^.

## Results

### Magnetic and electronic properties

**Fig. 1:**
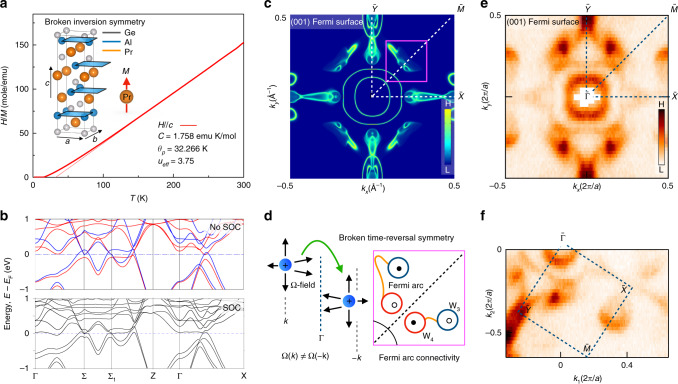
Lattice and electronic structure of non-centrosymmetric spin-orbit magnet PrAlGe. **a** Inverse magnetic susceptibility as a function of temperature (thick line) with fit to a Curie-Weiss law (thin line). Curie temperature *T*_C_ = 16 K was measured. Inset: crystal structure of PrAlGe in space group *I*4_1_*md* (No. 109). The square stacking pattern results in broken inversion symmetry. **b** Ab initio calculated bulk band structure of PrAlGe without (top panel) and with (bottom panel) spin-orbit coupling. The spin-up and spin-down states are shown in red and blue, respectively. **c** Ab initio calculated Fermi surface for the (001) surface. White dashed box: first quadrant of the surface BZ. **d** Left panel: breaking time-reversal symmetry allows the Weyl fermions (+), represented as sources of Berry curvature ***Ω*** fields, to be shifted in the crystal momentum space so they are no longer appear pairwise at  ±*k*. Right panel: Fermi arc connectivity (orange lines) schematic for the projected Weyl fermions (black and white circles), corresponding to the magenta box in **c**. A pair of projected W_3_ and W_4_ Weyl fermions on each side of the $\overline{\Gamma }-\overline{M}$ line. The predicted configuration of projected Weyl fermions manifestly breaks time-reversal symmetry. **e** Symmetrized and **f** un-symmetrized Fermi surface obtained by low-energy ARPES.

PrAlGe crystallizes in a body-centered tetragonal Bravais lattice with space group *I*4_1_*md* (No. 109). The basis consists of two Pr, two Al and two Ge atoms, Fig. 1a inset. Along the (001) direction, each atomic layer is comprised of one element, and the layer is shifted relative to the one below by half a lattice constant in either the *x* or *y* direction. Single crystal X-ray diffraction suggests that our samples possess the correct lattice structure and lack inversion symmetry (Supplementary Table 1). Measurements of magnetic susceptibility as a function of temperature were fitted to the inverse Curie-Weiss law. The obtained positive Weiss constant indicates the presence of ferromagnetic interactions, Fig. 1a. A direct measurement, to be discussed below, shows that PrAlGe is ferromagnetic with Curie temperature *T*_C_ = 16 K. The ferromagnetic ground state arises from the spin-polarized *f*-electron states that are locally coupled and aligned along the *c* axis, rendering the conduction electron bands near the Fermi level spin-polarized. This is reflected in our ab initio band structure calculations without spin-orbit coupling (SOC), in which it also shows that PrAlGe has a semi-metallic profile (top panel: Fig. 1b). The inclusion of SOC interactions couples the spin-up and spin-down states and slightly perturbs the electronic bands (bottom panel: Fig. 1b). The absence of inversion and time-reversal symmetry both contribute to band-splitting at generic crystal momenta. Kramers degeneracy splitting is linked to magnetism in the crystal. Of the Weyl fermions predicted in ferromagnetic PrAlGe^14^, two groups (labeled W_3_ and W_4_) are within  ±20 meV of the Fermi level. The ab initio calculated Fermi surface for ferromagnetic PrAlGe predicts the presence of topological Fermi arcs in each quadrant, with an asymmetry across the $\overline{\Gamma }-\overline{M}$ surface high-symmetry line, Fig. 1c, d. The Fermi arc asymmetry is connected to the absence of both inversion and time-reversal symmetry in PrAlGe (Supplementary Figs. 1–3).

### VUV-ARPES study of the (001) surface electronic structure

**Fig. 2:**
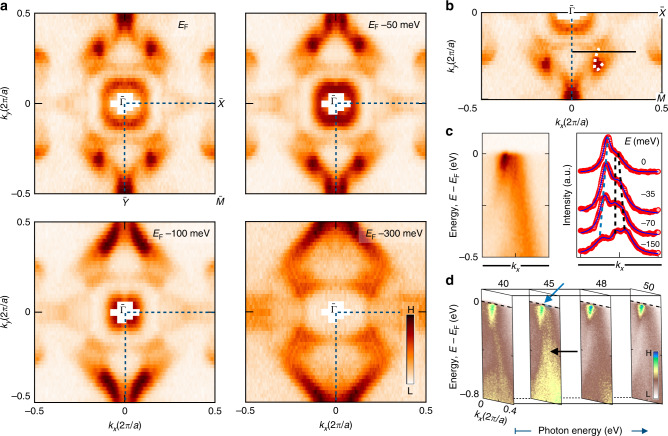
Fermiology and topology of the (001) surface electronic structure in PrAlGe. **a** Low-energy ARPES-measured Fermi surface and constant binding energy contours obtained with incident photon energies of 50 eV at *T* ≈ 11K. Blue dashed line: one quadrant of the surface Brillouin zone. **b** ARPES-measured Fermi surface with guides to the eye (white dashed line) tracking the “U” shaped candidate topological Fermi arc state. **c** Left: energy-momentum cut and, right: MDCs fitted at different binding energies with Lorentzian functions to track the candidate arc (blue dashed line) and bulk states (black dashed lines). The corresponding path is shown in **b**. **d** Photon-energy dependent ARPES along the horizontal line in **b**. Negligible *k*_*z*_ dispersion is observed for the candidate “U” shaped topological Fermi arc (blue arrow). Strong photon-energy dependence is observed for other states nearby (black arrow), suggesting that they are bulk states.

Motivated by our ab initio calculations and transport measurement observing ferromagnetism, we use angle-resolved photoemission spectroscopy (ARPES) measurements at low-photon energies (VUV-ARPES) to map the band structure of PrAlGe on the (001) surface at temperature *T* = 11 K, below *T*_C_. We observe that the ab initio calculation is qualitatively consistent with the measured Fermi surface, Fig. 1e, f. On constant-energy contours of varying binding energy, we observe the following dominant features, Fig. 2a: two concentric closed contours around the $\overline{\Gamma }$ point, a distinct “U” shaped state (marked by a guide to the eye, Fig. 2b), and additional surface states near the $\overline{Y}$ of the surface BZ. The inner closed concentric contour shrinks with deeper binding energy, showing a clear electron-like behavior. To better understand the nature of the “U” state and the spectral intensity in its vicinity, we study an energy-momentum cut through this state, Fig. 2c. We plot the Lorentzian-fitted momentum distribution curves (MDCs) at different binding energies and find that the “U” state disperses towards the Fermi level while a nearby band approaches *E*_F_ and then turns back toward deeper binding energies. We also find that the “U” state exhibits negligible photon energy dependence, suggesting that it is a surface state (Fig. 2d, Supplementary Fig. 4). A comparison with the ab initio calculated Fermi surface further suggests that the “U” state corresponds to the predicted Fermi arc connecting W_3_ and W_4_. The surface state nature of the “U” state and its correspondence with calculation suggests that the ARPES-measured state is a topological Fermi arc.

### Topological Fermi arcs and projected chiral charges

**Fig. 3:**
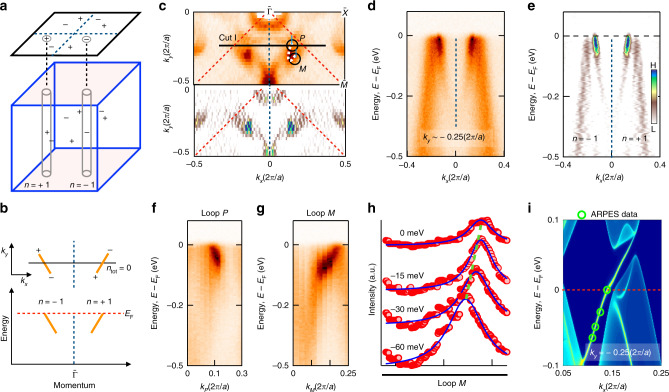
Observation of topological Fermi arcs and chiral charges in PrAlGe. **a** Bulk and surface Brillouin zone (BZ) with the Weyl fermions (±) and manifolds with Chern number *n*^32^. A closed loop enclosing the projected chiral charge in the surface corresponds to a cylinder in the bulk enclosing the Weyl fermion. **b** Top: topological Fermi arcs (orange) connecting the projected Weyl fermions carrying chiral charge  ±1. Bottom: a cut across two arcs (along the black line in top panel) with chiral edge modes (orange lines). **c** Top panel: Fermi surface obtained by ARPES at *T* ≈ 11K. Loop cuts of interest (*P* and *M*) are illustrated with black loops with the starting/end points marked by the vertical blue line. Bottom panel: 2D curvature plot of the above Fermi surface. **d** Measured band dispersion along horizontal Cut I. Blue dashed line: indicates the mirror symmetry. **e** Second-derivative plot of **d**. **f** ARPES-measured band dispersion along the loop *P*. Loop *P* encloses the termination point of the measured Fermi arc and shows a single left-moving chiral mode, corresponding to an enclosed Chern number *n* = −1. **g** Band dispersion along loop *M*. The observed right-moving chiral mode shows an enclosed Chern number *n* = +1. **h** Stack of MDCs along loop *M* at different binding energies, with Lorentzian fits. Green dashed line: guide to the eye tracking the peaks. **i** Calculated energy dispersion along *k*_*y*_ = −0.25 (2*π*/*a*) with the result from our ARPES spectra overlaid (open green circles).

To further explore the Fermi arc candidate we search for direct spectroscopic signatures of chiral charges in PrAlGe, taking advantage of the bulk-boundary correspondence between bulk Weyl fermions and surface Fermi arcs^11,32^. We study chiral edge modes along straight and loop energy-momentum cuts (Fig. 3a, b), and present a two-dimensional curvature plot of the measured Fermi surface to further highlight the momentum space trajectory of the Fermi arc candidate, Fig. 3c. A horizontal momentum cut at *k*_*y*_ ≈ −0.25 (2*π*/*a*) passes through a pair of “U” states, Fig. 3c, d. We observe signatures of a left-moving and right-moving mode related by mirror symmetry. A second derivative plot of the dispersion map further confirms the observed modes and suggests additional neighboring bulk bands which approach *E*_F_ and then turn back towards deeper binding energies, Fig. 3e. A comparison of this spectrum with the locations of the predicted Weyl fermions suggests that we can interpret the left- and right-moving modes as two chiral edge modes, associated with Chern number *n* = ±1, Fig. 3b. In this way, the momentum cut is associated with a 2D momentum-space slice carrying Chern number *n*_tot_ = 0, since *n*_l_ = −1 and *n*_r_ = +1. This again suggests that the left- and right-moving modes giving rise to two mirror partnered “U” states are topological Fermi arcs.

To provide further evidence of chiral charges in PrAlGe, we next perform an analysis of edge modes along closed loops in the surface BZ (black dashed circles labelled *P* and *M*, Fig. 3c). By counting chiral edge modes on these circular paths, we search for evidence of *n*_tot_ ≠ 0. Unrolling the energy-momentum dispersion for loop *P*, we observe one left-moving chiral mode that is dispersing towards *E*_F_, Fig. 3f. This result unambiguously shows chiral charge −1 on the associated bulk manifold. Analogously, along loop *M* we observe a right-moving chiral mode dispersing towards *E*_F_, Fig. 3g. By Lorentzian fitting of the MDCs along loop *M* at varying binding energies, we again observe a right-moving mode, Fig. 3h, suggesting that the corresponding bulk manifold encloses chiral charge  +1. As an additional check, the ab initio band dispersion calculation along *k*_*y*_ = −0.25 (2*π*/*a*) shows a right-moving chiral mode dispersing toward *E*_F_, Fig. 3i. An overlay of the Lorentzian fits of the chiral mode on the calculated band dispersion shows a match between our results (Supplementary Fig. 5). In this way, our low-energy ARPES spectra directly resolve topological Fermi arcs and demonstrate chiral charges in PrAlGe through the bulk-boundary correspondence^11,32^. Further, the observed Fermi arc asymmetry across the $\overline{\Gamma }-\overline{M}$ is consistent with our ab initio calculations for PrAlGe (Supplementary Fig. 2).

### Bulk Weyl cone dispersion

**Fig. 4:**
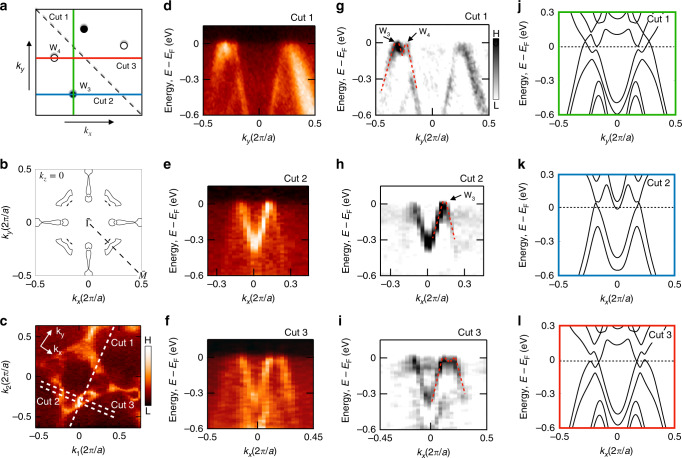
Observation of bulk Weyl cones in PrAlGe. **a** Distribution of equal but opposite chiral charged Weyl fermions (black and white circles) in the right-most (first) quadrant of the surface BZ. All other Weyl fermions are related by mirror symmetry along *k*_*x*_ = 0 and *k*_*y*_ = 0. The paths of interest are labeled as Cut 1 (green vertical line), Cut 2 (blue horizontal line), and Cut 3 (red horizontal line). **b** Ab initio calculated bulk Fermi surface (*k*_*x*_, *k*_*y*_) at *k*_*z*_ = 0. Symmetric across the $\overline{\Gamma }-\overline{M}$ high-symmetry line (black dashed line) are the Weyl cones bulk Fermi pockets in each quadrant. **c** SX-ARPES-measured Fermi surface on the *k*_*z*_ = 0 plane with incident photon energy 478eV and *T* < *T*_C_. The paths of interest (Cut 1–3; white dashed lines) correspond to those in **a**. **d**–**f** Energy-dispersion maps along Cut 1–3 with corresponding (**g**–**i**) second derivative and (**j**–**l**) ab initio calculation plots. The red dashed lines are guides to the eye for the Weyl cones.

Next, we provide a comparison between the experimental bulk band structure obtained by ARPES at soft X-ray energies (SX-ARPES) and our ab initio calculations (Fig. 4). To demonstrate the linear dispersion of the bulk Weyl cones, our analysis looks at the energy-dispersion maps along various horizontal and vertical paths that intersect the ab initio calculated positions of the Weyl fermions on the *k*_*z*_ = 0 plane, Fig. 4a. The large probing depth provided by SX-ARPES allows for a comparison between the ab initio calculated (Fig. 4b) and experimentally measured (Fig. 4c) bulk Fermi surface on the *k*_*z*_ = 0 plane. Both are qualitatively consistent and show an absence of asymmetry across the $\overline{\Gamma }-\overline{M}$ line. Along vertical cut 1 (green line: Fig. 4d) and horizontal cut 2 (blue line: Fig. 4e), we observe the linear dispersion of the W_3_ and W_4_ Weyl cones. Horizontal cut 3 (red line: Fig. 4f) further confirmes the linear dispersion of the W_4_ Weyl fermion. Second derivative plots of Cuts 1–3 with guides to the eye for the Weyl cones further illustrate their linear dispersion (Fig. 4g–i). Within the resolution of our measurements, the W_3_ and W_4_ Weyl fermions are located on the *k*_*z*_ = 0 plane at (0.15,  −0.32) 2*π*/*a* and (0.13,  −0.22) 2*π*/*a*, respectively. Due to our SX-ARPES measurement temperature being comparable to the Curie temperature of our PrAlGe samples, and limited SX-ARPES energy resolution or spectral linewidth, our data did not clearly resolve the Zeeman splitting, suggesting that further experimental work is needed. Howbeit, the qualitative agreement between our SX-ARPES measurements (Fig. 4d–i) and ab initio calculations (Fig. 4j–l) suggests the observation of bulk Weyl cones in PrAlGe.

### Anomalous Hall transport

**Fig. 5:**
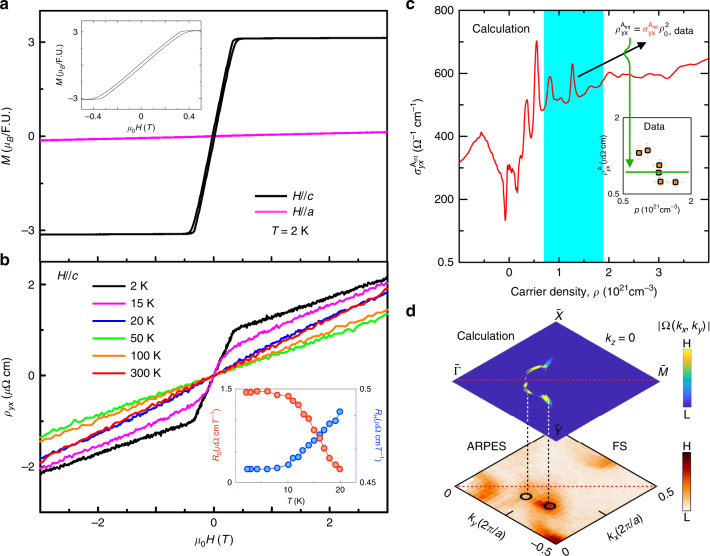
Observation of intrinsic anomalous Hall transport in PrAlGe. **a** Magnetization *M* of PrAlGe along the *c*-axis (black) and *a*-axis (magenta) versus magnetic induction *μ*_0_*H*. The Curie temperature is observed to be *T*_C_ =  16 K. Inset: zoom-in showing a hysteresis loop. **b** Hall resistivity *ρ*_*yx*_ as a function of *μ*_0_*H*. The inset shows the ordinary and anomalous Hall coefficients *R*_H_ (blue) and *R*_S_ (red) as a function of temperature extracted from the data. **c** Ab initio calculated intrinsic anomalous Hall conductivity ${\sigma }_{yx}^{{{{{\rm{A}}}}}_{{{{\rm{int}}}}}}$ as a function of carrier density. The shaded turquoise area corresponds to the carrier density of the measured PrAlGe samples. The inset shows the measured anomalous Hall resistivity ${\rho }_{yx}^{{{{\rm{A}}}}}$ as a function of carrier density p. The horizontal green line corresponds to the calculated intrinsic contribution to ${\rho }_{yx}^{{{{\rm{A}}}}}$. The error bars are set by the  ±10% error in measurement of the sample dimensions. **d** Surface-bulk-transport correspondence of Weyl fermions in PrAlGe. Top: Berry curvature magnitude ∣*Ω*(*k*)∣ at *k*_*z*_ = 0 summed over energies below the Fermi level, from ab initio calculation. Bottom: ARPES-measured Fermi surface, suggesting that the Berry curvature field is concentrated near the termination points of the Fermi arc and projection point of the Weyl cones (open circles) observed in ARPES.

Having experimentally demonstrated topological Fermi arcs and bulk Weyl cones in PrAlGe, we next investigate additional phenomena mediated by Berry curvature using magneto-transport (Supplementary Fig. 6). We study the magnetization *M* as a function of magnetic induction *μ*_0_*H* (Fig. 5a) and observe that PrAlGe is a soft ferromagnet with an easy axis along the *c*-direction. Furthermore, we find that the transverse resistivity *ρ*_*yx*_ exhibits an anomalous Hall effect, described by *ρ*_*yx*_ = *R*_H_*B* + *μ*_0_*R*_S_*M*, where *R*_H_ is the ordinary (Lorentz-force) Hall coefficient and *R*_S_ is the anomalous Hall coefficient^33^. As shown in the inset of Fig. 5b, *R*_S_ (zero for high *T*) grows rapidly toward large values while the small value for *R*_H_ (almost invariant for different *T*) decreases very quickly below *T*_C_. The observed behavior for *R*_H_ and *R*_S_ suggests that the anomalous Hall effect arises near *T*_C_. The measured *R*_S_ coefficient reaches a saturation value of about 1.5 μΩ cm T^−1^ at 2 K, where it dominates the response^34,35^. To investigate the origin of the observed behavior for the anomalous Hall effect, we plotted the anomalous Hall contribution ${\rho }_{yx}^{A}$ as a function of carrier concentration *p* = 1/e*R*_H_, where e is the charge of an electron. The result shows clustered values in the vicinity of 1 μΩcm for different samples, see Fig. 5c inset. To better understand the origin of ${\rho }_{yx}^{{{{\rm{A}}}}}$, we calculated the Berry curvature contribution to the anomalous Hall conductivity, the so-called intrinsic anomalous Hall conductivity, ${\sigma }_{yx}^{{{{{\rm{A}}}}}_{{{{\rm{int}}}}}}$ as a function of carrier concentration, Fig. 5c. The calculation predicts a roughly carrier concentration-independent value of ~600 Ω^−1^ cm^−1^ with carrier densities from *p* = 0.9 to 1.7 × 10^21^ cm^−3^. This corresponds to an intrinsic contribution to the anomalous Hall resistivity of ${\rho }_{yx}^{{{{{\rm{A}}}}}_{{{{\rm{int}}}}}}={\sigma }_{yx}^{{{{{\rm{A}}}}}_{{{{\rm{int}}}}}}{\rho }_{0}^{2}\approx 0.6\;{{{\mathrm{\mu}}}} \Omega$ cm, which we plot as a horizontal green line in inset, Fig. 5c. We find a remarkable agreement with the measured ${\rho }_{yx}^{{{{\rm{A}}}}}\approx 1\mu \Omega$ cm. This agreement suggests that the Berry curvature dominates the anomalous Hall response in PrAlGe.

### Bulk-boundary-transport correspondence in PrAlGe

To study the origin of the Berry curvature fields giving rise to the intrinsic anomalous Hall response, we compare the ARPES-measured Fermi surface with the calculated Berry curvature field. By summing over energies below *E*_F_, the Berry curvature magnitude ∣Ω(*k*)∣ at *k*_*z*_ = 0 shows concentrated hot spots, see Fig. 5d. The points of concentrated Berry curvature correspond to the position of the W_3_ and W_4_ Weyl fermions. A qualitative comparison with the ARPES-measured Fermi surface shows that within our momentum-space resolution the hot spot region coincides with the termination points of the measured topological Fermi arc. According to ref. ^36^, the AHE of an ideal magnetic Weyl semimetal with only one pair of Weyl fermions near the Fermi level can be written as: *σ*_Weyl_ = e^2^*k*/2*πh*, where *k* is their momentum space separation. A quantitative estimate using the measured Weyl fermion separation *k*_Weyl_ ≈ 0.15 Å^−1^ yields an intrinsic anomalous hall conductivity *σ*_Weyl_ ≈ 738 Ω^−1^ cm^−1^, consistent with our ab initio calculations. Collectively, our results provide strong evidence suggesting that the measured intrinsic anomalous Hall response arises from the Berry curvature contributions of the Weyl fermions in magnetic non-centrosymmetric PrAlGe^36^.

## Discussion

The captured surface- and bulk-states in our ARPES spectra and magneto-transport measurements, taken together with support from ab initio calculations, establish a surface-bulk-transport correspondence demonstrating that PrAlGe exhibits novel Berry curvature mediated topological electronic phenomena. Our results open new research directions in understanding and engineering tunable topological electronic properties in the non-centrosymmetric magnet PrAlGe. In particular, due to the absence of both time-reversal and inversion symmetry, exotic types of photogalvanic effects may emerge^37–40^. Additionally, topological currents in PrAlGe may also allow for the development of all-electrical spin generation and injection with no entropy production. Lastly, the soft-ferromagnetism may allow the spin-polarized topological currents to be turned on/off via an external magnetic field^41,42^. The control versatility and potential access to a large number of exotic phenomena makes magnetic non-centrosymmetric PrAlGe an exciting material platform for probing topological and quantum matter physics.

## Methods

### Single crystal growth

Single crystals of PrAlGe were grown by the self-flux technique with purified Pr ingots (99.9%), Al shots (99.99%), and Ge pieces (99.99%). Stoichiometric mixtures of Pr1Al18Ge1 were set in alumina crucibles and then sealed in fused silica ampoules under partially filled argon atmosphere. After being pegged at 1150 °C for a few hours, the ampoules were slowly cooled down to the centrifugal temperature 750 °C at a rate of 0. 1 °C/min.

### Angle-resolved photoemission spectroscopy

Low-energy ARPES measurements (VUV-ARPES) were carried out at Beamlines (BL) 5–2 of the Stanford Synchotron Radiation Lightsource (SSRL) at SLAC in Menlo Park, CA, USA, with a Scienta R4000 electron analyzer. The angular resolutions was better than 0.2°, and the energy resolution was better than 20 meV. The beam spot size was about 20 × 40 μm^2^. Samples were cleaved in situ and measured under vacuum better than 5 × 10^−11^ Torr and temperatures <11 K. Soft X-ray ARPES measurements (SX-ARPES) were performed at the ADRESS beamline at the Swiss Light Source in the Paul Scherrer Institut (PSI) in Villigen, Switzerland. The combined (beamline and analyzer) energy resolution of the SX- ARPES measurements varied between 40 and 80 meV. The angular resolution of the SX- ARPES analyzer was better than 0.2°. PrAlGe samples were cleaved in situ under a vacuum condition better than 5 × 10^−11^ Torr and temperature less than the Curie temperature *T*_C_.

### Magnetization measurements

Magnetization measurements were performed using the Quantum Design Magnetic Property Measurement System (MPMS-3).

### Transport measurements

Resistance and Hall effect measurements were performed in a Quantum Design Physical Property Measurement System (PPMS), using the standard four-probe technique with silver paste contacts that were cured at room temperature.

### First-principles calculations

The first-principles calculations were performed within the density functional theory (DFT) framework using the projector augmented wave method as implemented in the VASP package^43,44^. The generalized gradient approximation (GGA) was used^45^ for the exchange-correlation effect and the Hubbard energy *U* used in the calculation is 4 eV. A Γ-centered *k*-point 14 × 14 × 14 mesh was used and spin-orbit coupling (SOC) was included in self-consistent cycles. To generate the (001)-surface states of PrAlGe, Wannier functions were generated using the *d* and *f* orbitals of Pr, and the *s* and *p* orbitals of Al and Ge. The surface states were calculated for a semi-infinite slab by the iterative Green’s function method. They were optimized based on experimental results^46^.

## Supplementary information

Supplementary Information

## Data Availability

The data supporting the findings of this study are available within the paper, and other findings of this study are available from the corresponding author upon reasonable request.
