# Author Correction: Observation of Weyl fermions in a magnetic non-centrosymmetric crystal

**DOI:** 10.1038/s41467-022-29127-5

**Published:** 2022-03-11

**Authors:** Daniel S. Sanchez, Guoqing Chang, Ilya Belopolski, Hong Lu, Jia-Xin Yin, Nasser Alidoust, Xitong Xu, Tyler A. Cochran, Xiao Zhang, Yi Bian, Songtian S. Zhang, Yi-Yuan Liu, Jie Ma, Guang Bian, Hsin Lin, Su-Yang Xu, Shuang Jia, M. Zahid Hasan

**Affiliations:** 1 Laboratory for Topological Quantum Matter and Advanced Spectroscopy (B7), Department of Physics, Princeton University, Princeton, NJ 08544 USA; 2 International Center for Quantum Materials, School of Physics, Peking University, Peking, China; 3 Rigetti Computing, Berkeley, CA 94720 USA; 4 Key Laboratory of Artificial Structures and Quantum Control, School of Physics and Astronomy, Shanghai Jiao Tong University, Shanghai, China; 5 Department of Physics and Astronomy, University of Missouri, Columbia, MO USA; 6 Institute of Physics, Academia Sinica, Taipei, 11529 Taiwan; 7 Collaborative Innovation Center of Quantum Matter, 100871 Beijing, China; 8 Princeton Institute for Science and Technology of Materials, Princeton University, Princeton, NJ 08544 USA; 9 Lawrence Berkeley National Laboratory, Berkeley, CA 94720 USA

**Keywords:** Theory and computation, Condensed-matter physics

Correction to: *Nature Communications* 10.1038/s41467-020-16879-1, published online 03 July 2020.

The original version of this Article omitted the following from the Acknowledgements: ‘The soft X-ray photoemission data presented in Fig. 4 was collected at the ADRESS beamline of the Swiss Light Source^47,48^. The authors acknowledge synchrotron radiation beamtime at the ADRESS beamline of the Swiss Light Source of the Paul Scherrer Institut in Villigen, Switzerland under Proposal 20170898. Specifically, we acknowledge the important contributions of Vladimir N. Strocov, Alla Chikina, and Niels B. M. Schröter in the acquisition of the soft X-ray ARPES spectra’. This has now been corrected in both the PDF and HTML versions of the Article.

The original version of this Article omitted two references to previous works. These have been added as Reference 47 and Reference 48 in the Acknowledgments: ‘The soft X-ray photoemission data presented in Fig. 4 was collected at the ADRESS beamline of the Swiss Light Source^47,48^’. This has been corrected in the PDF and HTML versions of the Article.
