# Supplementary material for: Observation of Weyl fermions in a magnetic non-centrosymmetric crystal
Source: Nat Commun. 2020 Jul 3;11:3356. doi: 10.1038/s41467-020-16879-1 (PMC7335064; doi:10.1038/s41467-020-16879-1)
Supplement: Supplementary file 1 — Supplementary Information [file 41467_2020_16879_MOESM1_ESM.pdf]

Supplementary Information

Observation of Weyl fermions in a magnetic non-centrosymmetric crystal

Sanchez et al.

Supplementary Note 1: Single crystalline X-ray diffraction. The lattice structure of PrAlGe was determined by using single-crystalline X-ray diffraction, Bruker APEX-II. Full data sets were collected for θ ranging from $6.04° - 67.82°$ at temperature 300 K, and a total of 1261 reflections were obtained. The tetragonal crystal symmetry of $I4_1md$ were refined using the SHELX97-97 programs[1].

F. W. (g/mol)	481.04
Crystal System	Tetragonal
Space Group	$I4_1md$ (No. 109)
a(Å)	4.2452(16)
c(Å)	14.6420(60)
V(Å^3)	263.9(2)
Density (g/cm^3)	4.19
Temperature (K)	293
λ (Å)	1.54178
No. Reflections	1261
θ-range (deg)	6.04 - 67.82
R_f	0.0892
Goodness of Fit	2.407

Supplementary Table 1: Single crystalline X-ray diffraction on PrAlGe at 300K.

Supplementary Note 2: Bulk band-structure of non-magnetic and ferromagnetic PrAlGe. According to ab initio calculations, the paramagnetic band structure of PrAlGe is quite metallic, with the flat f-electrons slightly above the Fermi level, Supplementary Figure 1**a**. In the ferromagnetic state, the f-electrons are pushed far below the Fermi level ($E \approx -2.8$eV), Supplementary Figure 1**b**. The local moments of the f-electrons serve as an effective Zeeman field that renders the conduction bands (s, p, d orbitals) spin polarized. Note, PrAlGe is semi-metallic in its ferromagnetic state, which is consistent with our ARPES measurements.

Supplementary Note 3: Bulk bands and Fermi arcs of non-magnetic LaAlGe and ferromagnetic PrAlGe. A difference in the electron orbital occupation of Pr relative to La is that Pr

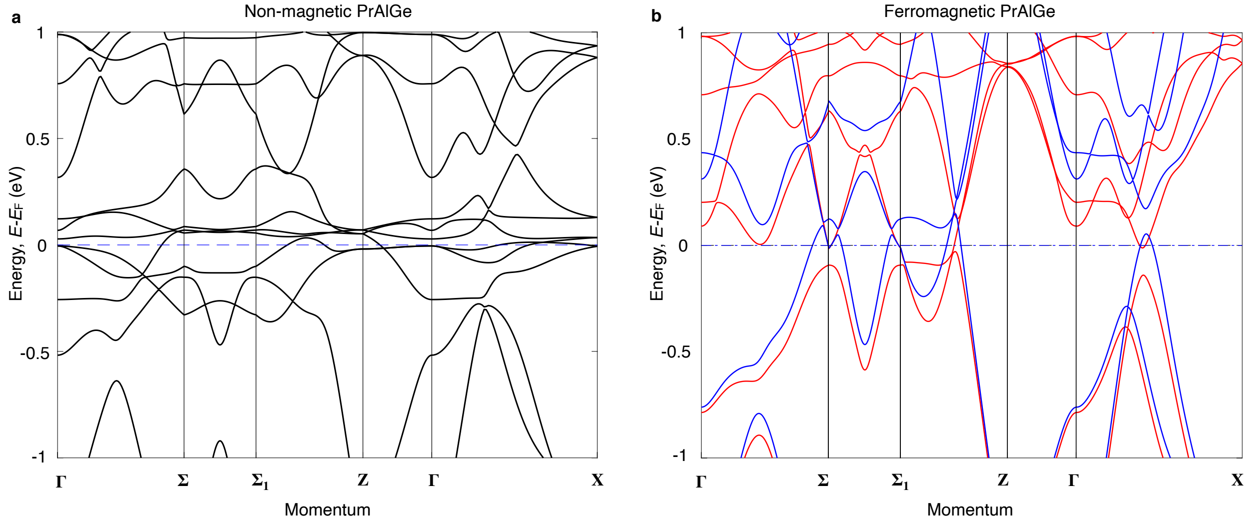

Supplementary Figure 1: **Bulk-band structure of non-magnetic and ferromagnetic PrAlGe.** **a**, Non-magnetic PrAlGe and **b**, Ferromagnetic PrAlGe in the absence of spin-orbit coupling.The spin-up and spin-down states are shown in red and blue, respectively.

contains one additional f-electron. According to our ab initio calculations, the coupling between the f-electrons' local moments leads to ferromagnetism in PrAlGe. The local moments serve as an effective Zeeman field, making the conduction (s, p, d orbitals) bands spin polarized, see Supplementary Figures 2**a**,**b**. The flat-f bands in ferromagnetic PrAlGe are shifted to $E \approx -2.8$eV. Both have a semi-metallic electronic profile.

The local moments in PrAlGe have further effects. One effect is that of creating a new magnetic Weyl fermion (W_4) in the momentum space of PrAlGe. Consequently, the Fermi arc connectivity between non-magnetic LaAlGe and ferromagnetic PrAlGe differ in a significant manner. Due to the W_4 Weyl fermions in the ferromagnetic state of PrAlGe, the Fermi arc connectivity no longer extend across the $\bar{\Gamma} - \bar{M}$ surface high-symmetry line and thereby showing different chiral charge distributions, see Supplementary Figures 3**a**,**b**. Using VUV-ARPES, we resolved a Fermi arc surface state extending along one side of the $\bar{\Gamma} - \bar{M}$ surface high-symmetry line and therefore demonstrating the aforementioned asymmetry, see Supplementary Figure 3**c**. In this sense, our surface-sensitive ARPES measurements demonstrate evidence of ferromagnetism and its effect on the Fermi arc connectivity in PrAlGe.

Supplementary Note 4: Photon-energy-dependent ARPES spectra. The chiral modes presented in the main text are shown in Supplementary Figure 4**a**. Photon-energy-dependent ARPES probes the variation of the band structure along the momentum direction that is perpendicular to

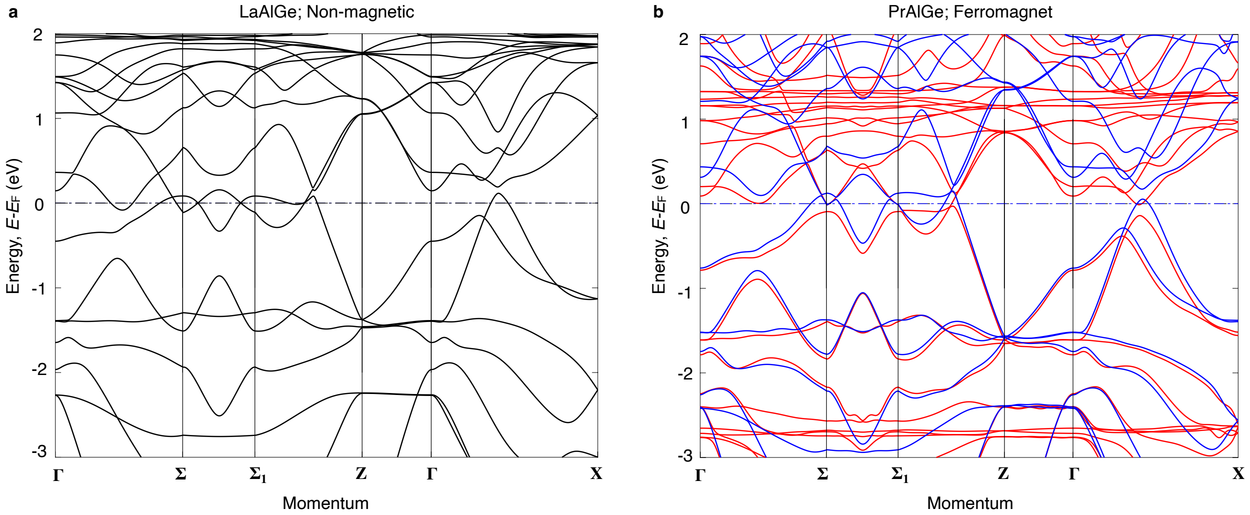

Supplementary Figure 2: **Bulk-band structure of non-magnetic LaAlGe and ferromagnetic PrAlGe. a**, Non-magnetic LaAlGe and **b**, Ferromagnetic PrAlGe in the absence of spin-orbit coupling. The spin-up and spin-down states are shown in red and blue, respectively.

the sample surface (the k_z direction in our geometry). Measurements were conducted by varying the photon energy from 30eV to 50eV, Supplementary Figure 4**b**. The dispersion of the bands at deeper binding energies depends drastically on the photon energy (k_z), whereas the dispersion of Fermi arcs near the Fermi level shows negligible dependence on k_z. This result demonstrates the surface state nature of the observed Fermi arcs.

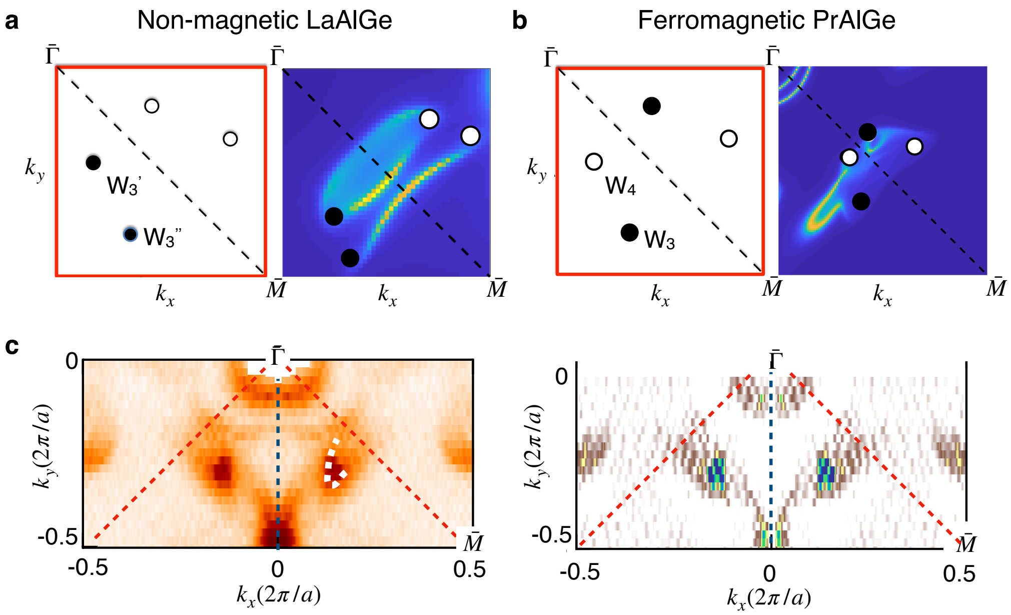

Supplementary Figure 3: **Fermi arc connectivity in non-magnetic LaAlGe and ferromagnetic PrAlGe.** **a**, Left panel: projected Weyl nodes on one quadrant of the FS[7]. Projected Weyl nodes with equal and opposite chiral charge are indicated by black and white circles. The surface high-symmetry line along $\bar{\Gamma} - \bar{M}$ is marked with a black dashed-line. Right panel: the calculated Fermi surface for non-magnetic LaAlGe shows Fermi arcs extending across $\bar{\Gamma} - \bar{M}$. **b**, Left panel: projected Weyl fermions in ferromagnetic PrAlGe, due to the emergence of W_4 Weyl fermions, have pairs of equal and opposite chiral charge on one half of the quadrant. Right panel: the calculated Fermi surface for PrAlGe shows that the Fermi arc no longer extending across the $\bar{\Gamma} - \bar{M}$ creates an asymmetry that directly results due to ferromagnetism. **c**, Left panel: ARPES measured Fermi surface. Right panel: corresponding 2D curvature plot for PrAlGe at measuring temperature $T < T_C$. Asymmetry across the $\bar{\Gamma} - \bar{M}$ line is observed for the Fermi arc surface state, consistent with theoretical calculations for ferromagnetic PrAlGe. The white dashed line is a guide to the eye for the Fermi arc.

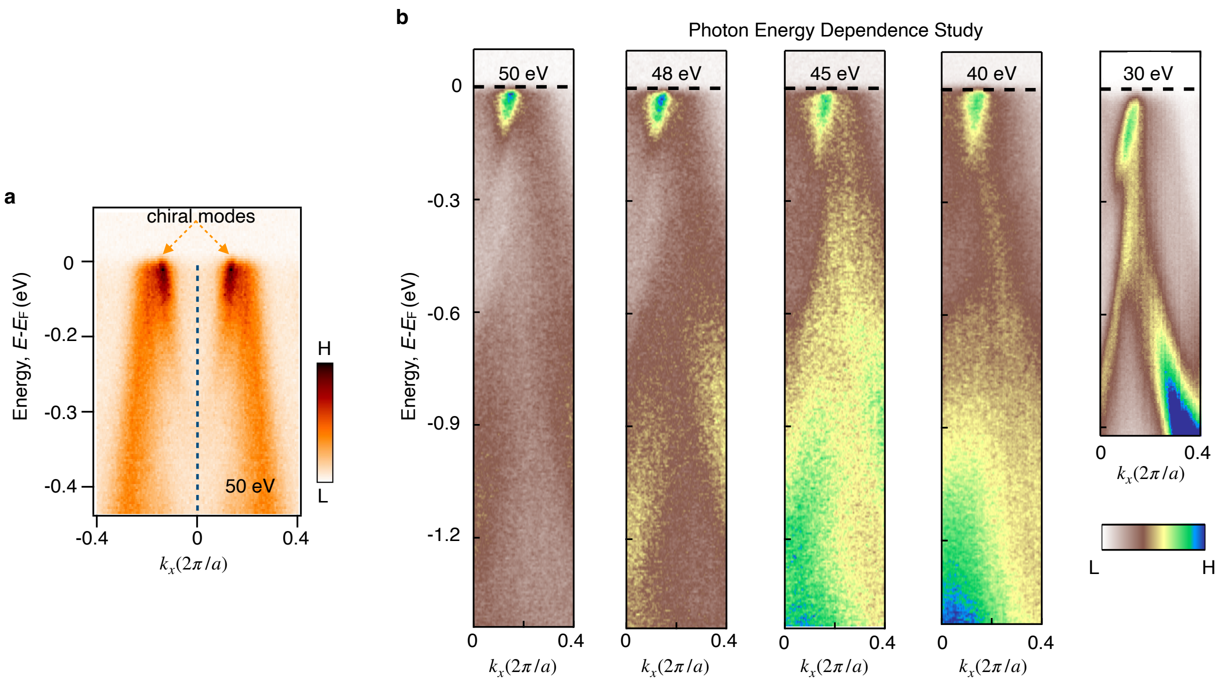

Supplementary Figure 4: **Photon-energy-dependent ARPES spectra.** **a**, Adapted from the main text. **b**, Photon-energy dependence study on the chiral modes. The right-most panel (30eV) was collected on a freshly cleaved PrAlGe crystal.

Supplementary Note 5: Tracking the chiral mode by fitting ARPES momentum distribution curves. The following procedure was performed to track the chiral mode as a function of binding energy, Supplementary Figure 5**a**,**b**. MDCs are collected for various energy values. Each MDC is fitted with Lorentzian functions to pinpoint the k_x value that corresponds to the peak maxima for the chiral mode, Supplementary Figure 5**c**-**f**. The MDC fitted peak maxima are then plotted on top of the ab initio energy-dispersion calculation along $k_y = -0.25(2\pi/a)$ and marked with green open circles, shown in Fig.3**i**.

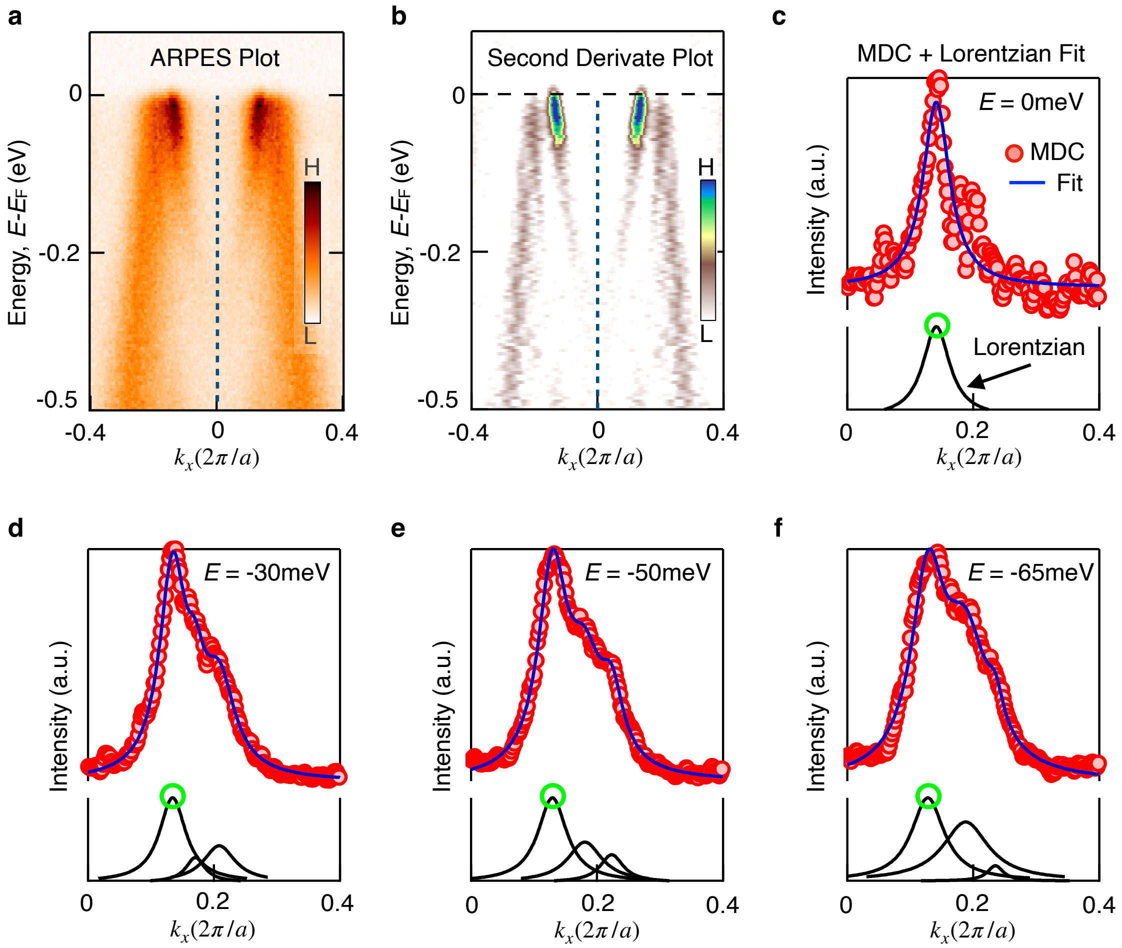

Supplementary Figure 5: **Tracking the chiral mode with MDCs. a**, ARPES measured energy-dispersion cut through a pair of Fermi arcs that are mirror symmetry related. Figure adapted from Fig.2d. **b**, Second-derivative plot of panel (a). Figure adapted from Fig.2e. **c**-**f**, MDC fitting procedure used to track the chiral mode as a function of binding energy. The intensity maxima corresponding to the chiral mode is indicated with an open green circle.

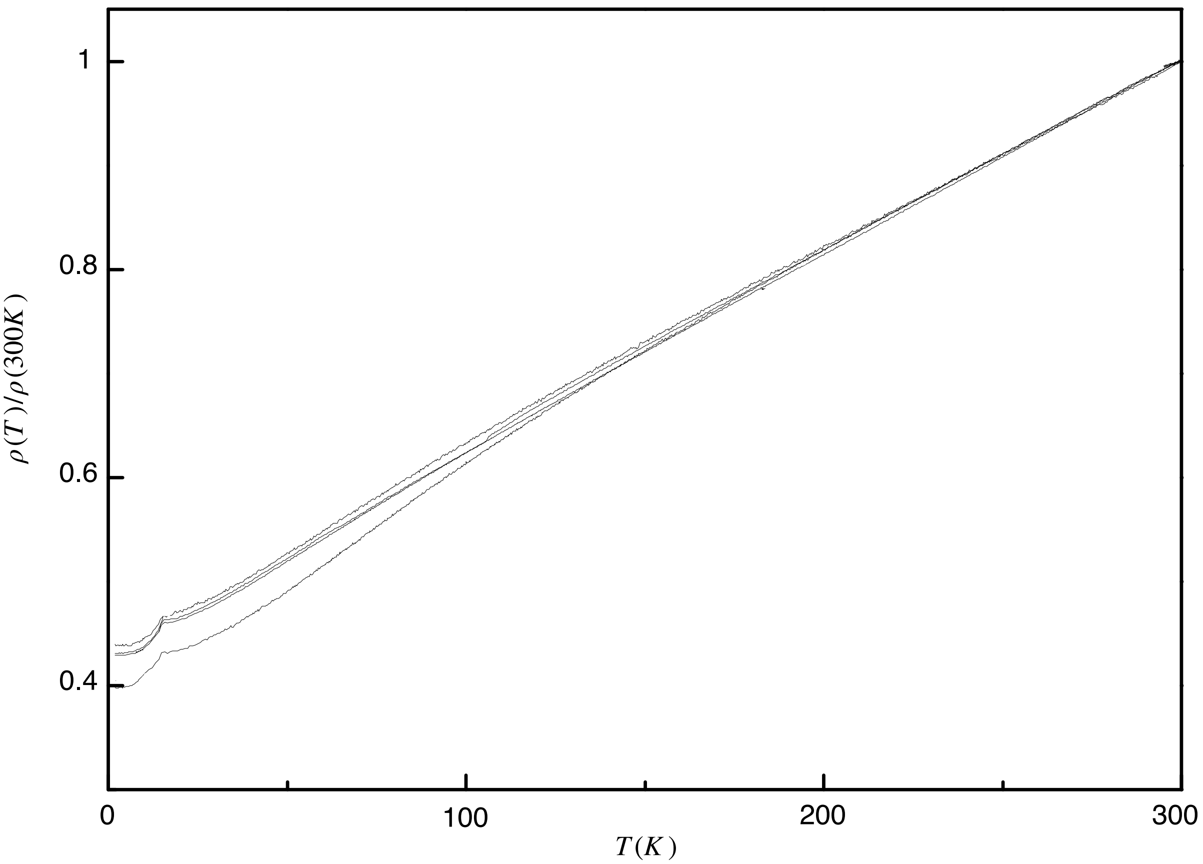

Supplementary Figure 6: **Metallic transport in PrAlGe.** The normalized temperature dependent resistivity $\rho(T)/\rho(300\text{K})$ for different samples is almost identical. A kink is clearly observed in the curves around T_{C}. The resistivity decreases as the temperature is lowered below T_{C} due to a loss in spin disorder scattering. Note that the decrease in resistivity below T_{C} is gradual, and very small for the entire temperature range.

Supplementary References

[1] Sheldrick, G. M. Acta Crystallogr., Sect. A: Found. Adv. A 64, 112 (2008).
